# Supplementary material for: Deep visual proteomics uncovers nociceptor diversity and pain targets
Source: Nat Commun. 2026 Apr 11;17:3437. doi: 10.1038/s41467-026-71418-8 (PMC13076600; doi:10.1038/s41467-026-71418-8)
Supplement: Supplementary file 1 — Supplementary Information [file 41467_2026_71418_MOESM1_ESM.pdf]

Supplementary Figures

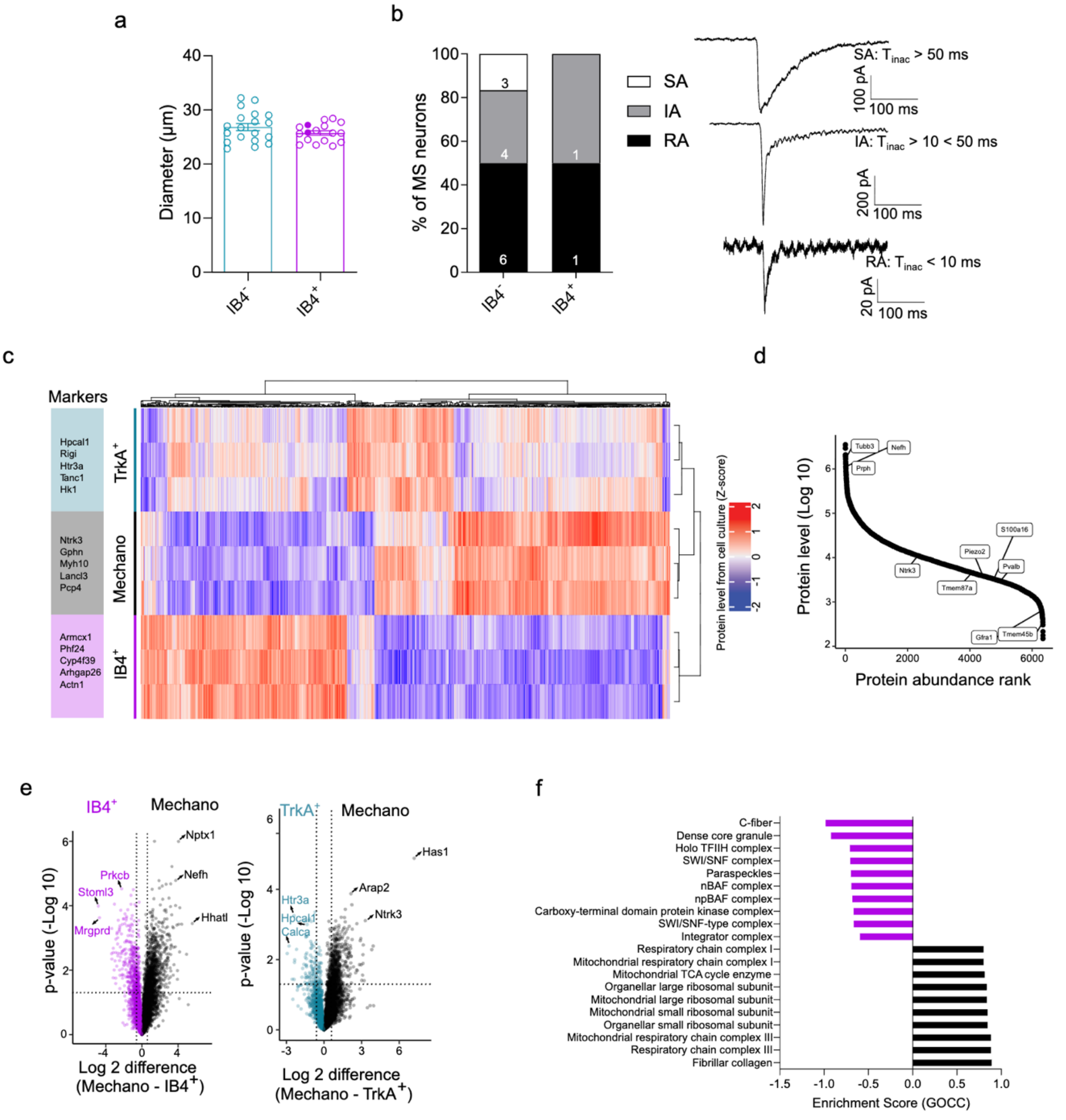

Supplementary Fig 1: Proteomics-based, subset-specific markers of sensory neurons. a) Bar plot showing the distribution of the diameters of patched nociceptors. Filled dots in IB4+ neurons represent the two mechanosensitive non-peptidergic neurons. $IB4^-$: n = 19, $IB4^+$: n = 17. **b)** Contingency plot of percent mechanically active currents with slowly adapting (SA), intermediately adapting (IA) and rapidly adapting (RA) biophysical properties along with their representative traces. **c)** Hierarchical clustering of proteins identified in cultured peptidergic and non-peptidergic nociceptors and mechanoreceptors. The top five proteins in each cluster are highlighted on the left. Each column represents z-scored protein levels. **d)** Dynamic range of protein abundance in the mechanoreceptor subset. **e)** Volcano plot showing the pairwise proteomic comparison between non-peptidergic (left) or peptidergic (right) nociceptors and mechanoreceptors with marker proteins highlighted. **f)** Pathway enrichment analysis based on

two sided t-test differences between non-peptidergic nociceptors and mechanoreceptors. The top ten pathways with a Benjamini-Hochberg false discovery rate (FDR) < 0.05 are shown. Cyan = peptidergic, magenta = non-peptidergic, black = mechanoreceptor. Error bars represent s.e.m. Source Data are provided as a Source Data file.

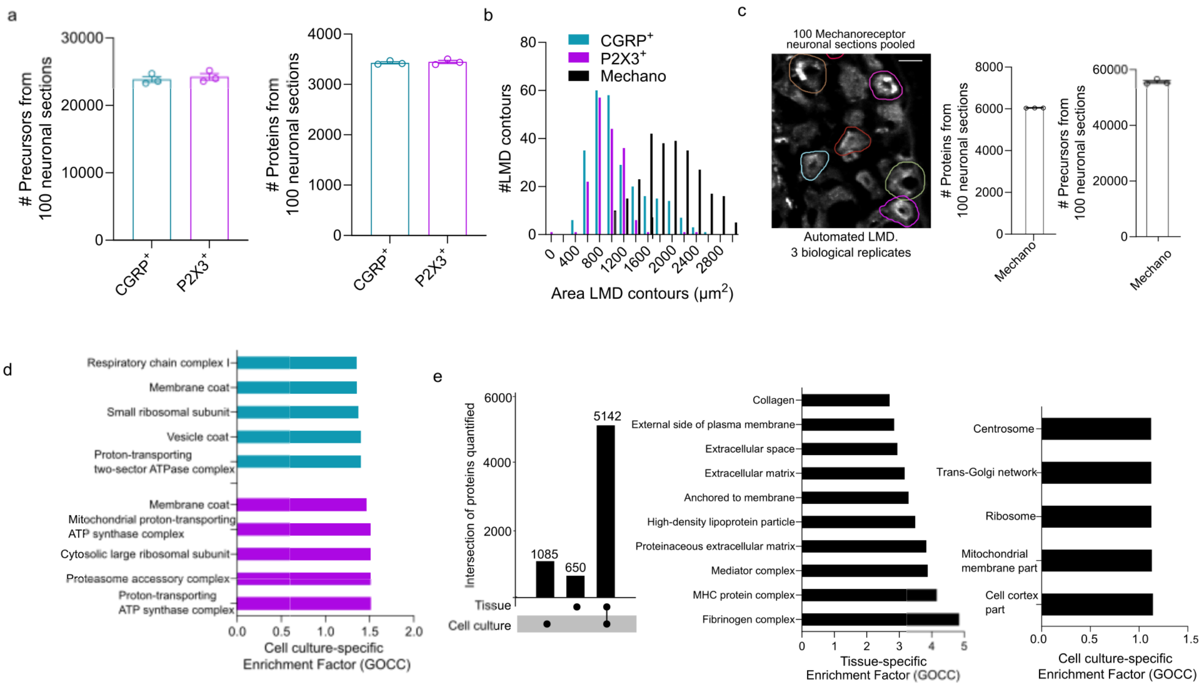

Supplementary Fig 2: Protein quantification from intact sensory neuron tissue sections. **a)** Number of precursors and proteins identified from peptidergic (P2X3$^-$/CGRP$^+$) and non-peptidergic (P2X3$^+$/CGRP$^-$) nociceptors. N = 3 mice. **b)** Histogram of area of the contours isolated by automated laser microdissection (LMD) from nociceptors and mechanoreceptors. **c)** Representative image of NF200 stained dorsal root ganglion (DRG) tissue sections with highlighted LMD contours (left). Scale bar = 20 μm. Number of precursors and proteins identified from the mechanoreceptors (right). N = 3 mice. **d)** Pathway enrichment analysis (Fischer's exact test) of uniquely identified proteins from cultured nociceptors. **e)** Upset plot showing common and unique proteins quantified from mechanoreceptors in intact tissue and in culture (left), along with pathway enrichment analysis (Fischer's exact test) of uniquely identified proteins from intact, tissue-derived (middle) and cultured (right) mechanoreceptors. Error bars represent s.e.m. Source Data are provided as a Source Data file.

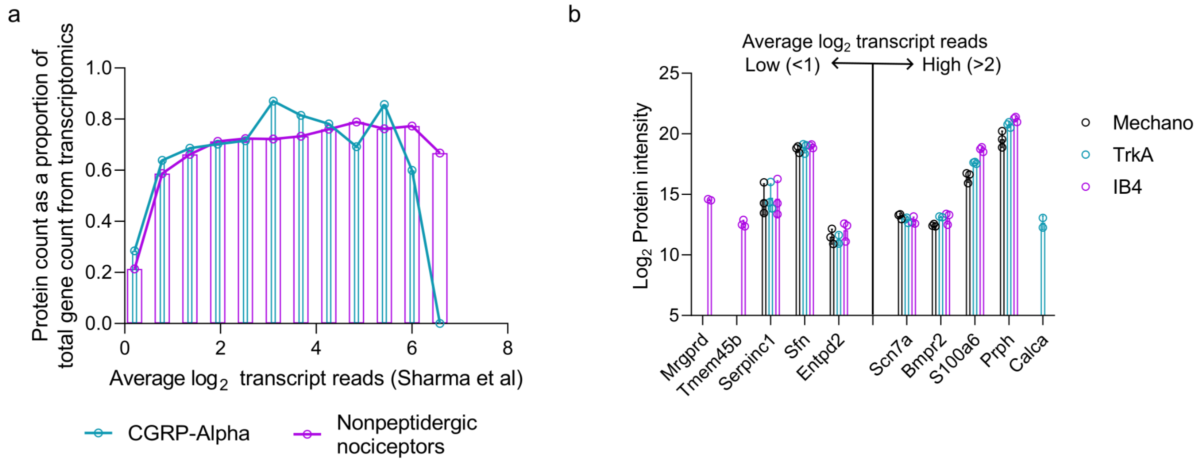

Supplementary Fig 3: Comparison of gene detection by transcriptomics and by proteomics. **a)** Histogram showing genes detected in proteomics as a fraction of genes detected using transcriptomics (from Sharma et al's scRNA seq dataset) across a range of log transformed average transcript reads. **b)** Plots of protein intensity for selected genes with low and high average transcript reads. Cyan = peptidergic, magenta = non-peptidergic, black = mechanoreceptor. Source Data are provided as a Source Data file.

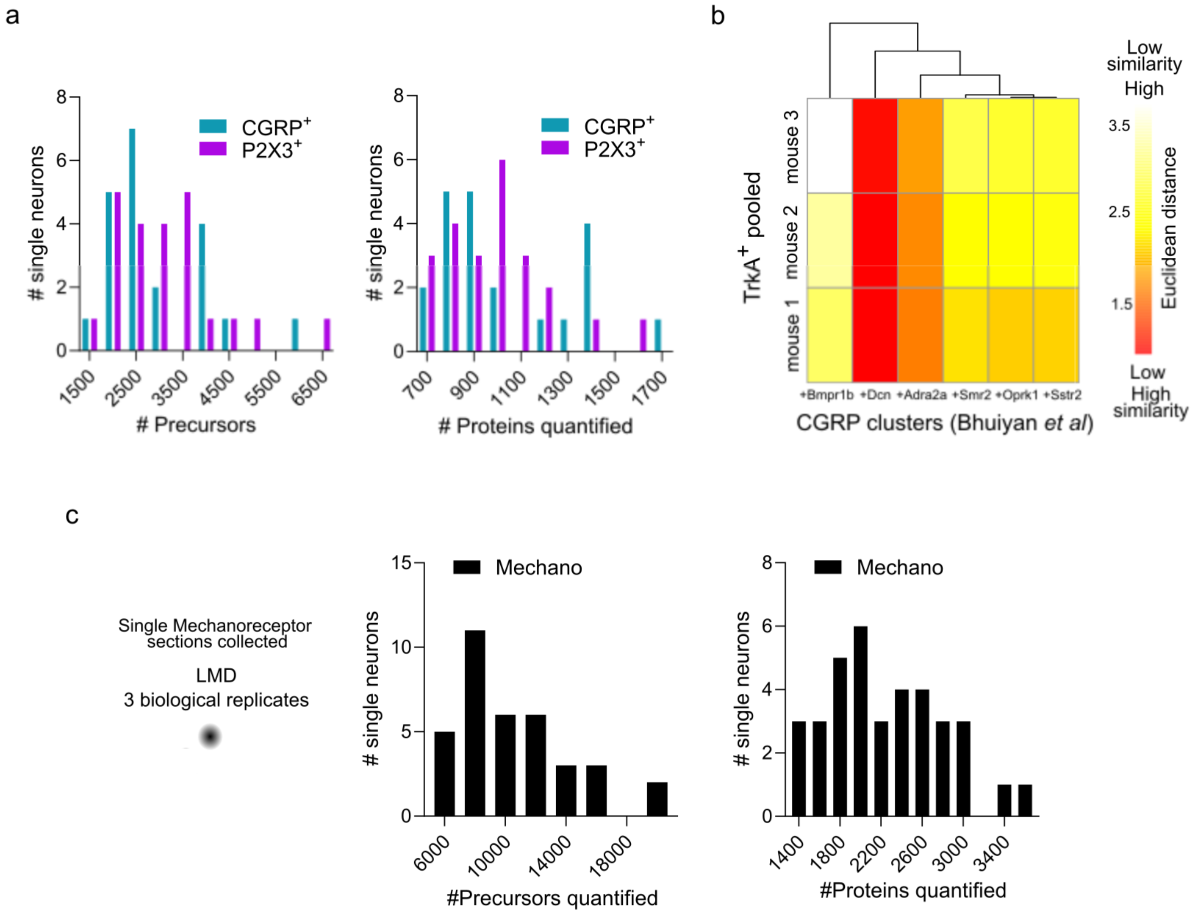

Supplementary Fig 4: Protein quantification from single sensory neuron sections. **a)** Histograms showing the number of precursors and protiens quantified from each nociceptor section. **b)** Similarity plot based on Euclidean distance between enriched genes in CGRP (peptidergic) subclusters (columns) identified by single-cell transcriptomics, and proteome of pooled, cultured TrkA^{+} peptidergic nociceptors (row). Red = high similarity; white = low similarity. **c)** Histogram showing the number of precursors and proteins quantified from each individual mechanoreceptor section. Source Data are provided as a Source Data file.

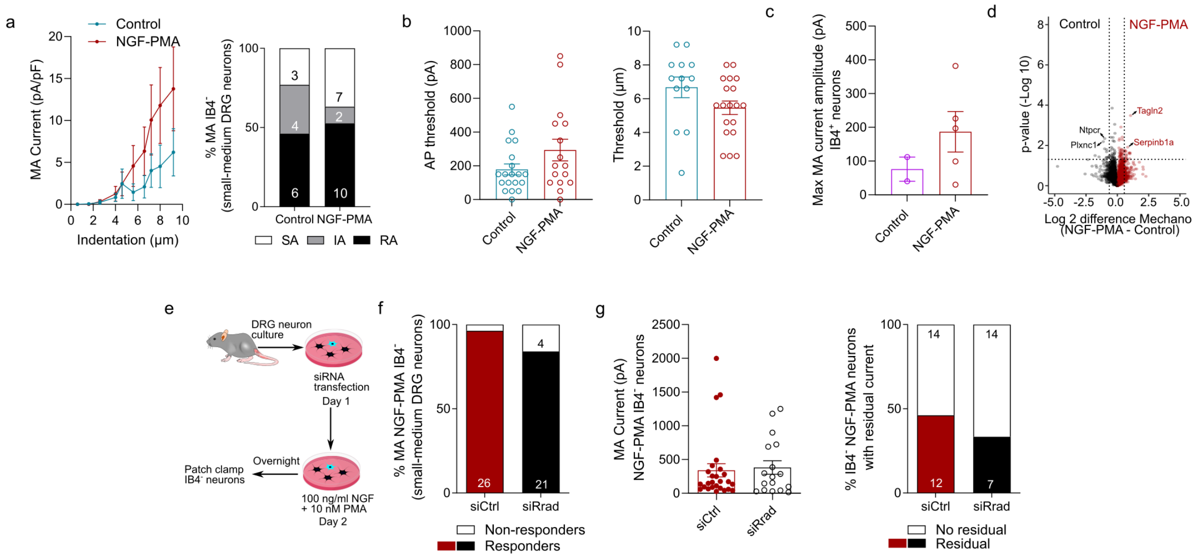

Supplementary Fig 5: Rrad does not ameliorate inflammation-induced mechanical hypersensitivity of peptidergic nociceptors. a) Mechanically active (MA) current amplitude at across a range of indentation in control and NGF-PMA sensitized peptidergic nociceptors, along with quantification of the proportion of slowly adapting (SA), intermediately adapting (IA) or rapidly adapting (RA) currents elicited. **b)** Action potential generation (control: n = 19, NGF-PMA: n = 16) and mechanical indentation threshold (control: n = 19, NGF-PMA: n = 16) of control (cyan) and NGF-PMA treated (red) peptidergic nociceptors. **c)** Bar graph showing indentation-evoked mechanosensitive current amplitudes of control (magenta, n = 2) and inflamed (red, n = 5) non-peptidergic nociceptors. **d)** Volcano plot showing the pairwise proteomic comparison between control and NGF-PMA treated mechanoreceptors with the top regulated proteins highlighted. **e)** Schematic workflow of the validation experiment by knockdown of Rrad using siRNA prior to NGF-PMA treatment and patch-clamp electrophysiology of peptidergic neurons. **f)** Percentage of mechanically sensitive NGF-PMA treated peptidergic nociceptors transfected with control siRNA (red) and Rrad-targeting siRNA (black). The number of neurons is indicated in the bars. **g)** Current amplitudes (left) and percentage of neurons with residual currents in NGF-PMA treated peptidergic nociceptors transfected with control siRNA (red) and Rrad-targeting siRNA (black). Error bars represent s.e.m. Source Data are provided as a Source Data file.
