# Supplementary material for: Deep visual proteomics uncovers nociceptor diversity and pain targets
Source: Nat Commun. 2026 Apr 11;17:3437. doi: 10.1038/s41467-026-71418-8 (PMC13076600; doi:10.1038/s41467-026-71418-8)
Supplement: Supplementary file 2 — Description of Additional Supplementary Files [file 41467_2026_71418_MOESM2_ESM.pdf]

Description of Additional Supplementary Files

File name: Supplementary Data 1

Description: Relative intensities of transmembrane proteins and ion channels identified in different subsets of sensory neurons.

File name: Supplementary Data 2

Description: Z-scored list of ANOVA significant proteins from sensory neuronal subsets.

File name: Supplementary Data 3

Description: Relative intensities (non-imputed, median normalized) of proteins identified from intact, pooled peptidergic and non-peptidergic nociceptors.

File name: Supplementary Data 4

Description: Relative intensities (non-imputed, median normalized) of proteins identified from single intact mechanoreceptors.

File name: Supplementary Data 5

Description: Relative intensities (non-imputed, median normalized) of proteins identified in single sections of nociceptors.

File name: Supplementary Data 6

Description: List of upregulated proteins after inflammation in peptidergic, non-peptidergic nociceptors and mechanoreceptors. List derived from the pairwise proteomic comparison between inflamed and control samples.
